# A dyadic advance care planning intervention for people with early-stage dementia and their family caregivers in a community care setting: a feasibility trial

**DOI:** 10.1186/s12877-023-03815-3

**Published:** 2023-03-01

**Authors:** Cheryl Chi-yan Yeung, Ken Hok-man Ho, Helen Yue-lai Chan

**Affiliations:** The Nethersole School of Nursing, Faculty of Medicine, The Chinese University of Hong Kong, 6-8/F, Esther Lee Building, Shatin, Hong Kong SAR China

**Keywords:** Advance care planning, Early-stage dementia, Feasibility, Dyadic

## Abstract

**Background:**

Advance care planning (ACP) is highly relevant for people with early-stage dementia to communicate their care preferences for serious illness conditions with their family caregivers before they become mentally incapacitated.

**Methods:**

A multi-centre, quasi-experimental study was conducted to test the feasibility and acceptability of a theory-guided, dyadic ACP intervention (‘Have a Say’ programme) among participants with early-stage dementia–family caregiver dyads. The feasibility of the trial design, intervention procedures, subject recruitment and retention, and study instruments were assessed. Study outcomes were measured at baseline (T0), immediately after the intervention (T1), and at 1 month (T2) and 3 months post-intervention (T3). Acceptability of the intervention was determined by the satisfaction score, completion rate and qualitative interviews as process evaluation with a purposive sample of participants and ACP facilitators. Generalised estimating equations were performed to examine differential changes between groups over time, with covariates adjusted.

**Results:**

Subject recruitment from five elderly community centres yielded a recruitment rate of 60% and resulted in 36 client–caregiver dyads. The intervention was acceptable to the dyads, with a mean satisfaction score of 4.4 out of 5 and completion rate of 94.4%. The attrition rates at T1, T2, and T3 were 8.3%, 13.9%, and 19.4%, respectively. The intervention group reported a significantly greater improvement in the readiness for ACP at T1, self-efficacy for ACP at T3, and dyadic concordance on end-of-life care preferences at all time points than the control group, but not on depressive symptoms. Family caregivers in the intervention group reported a significantly higher caregiving burden at T2 than the control group. The qualitative findings revealed that triadic involvement of and trusting relationships among the dyads and ACP facilitators, and documentation of clients’ views are the programme strengths, while the structured format and discussion about medical issues posed implementation challenges.

**Conclusions:**

This ACP intervention and trial design were feasible and acceptable to the dyads. Several refinements were identified, including adding a nurse-led group-based session for information giving, allowing flexibility in arrangement, and adding measure of ACP engagement of family caregivers. A rigorous trial to test the effects of the ACP intervention is warranted.

**Trial registration:**

Retrospectively registered on 14/08/2020 at clinicaltrials.gov (Identifier: NCT04513106).

## Background

Dementia is a progressively deteriorating and life-limiting disease commonly affecting older adults [1]. Over time, the care needs and dependency of people with dementia increase, while their decision-making capacity decreases. Most of them rely on their family members to make surrogate decisions regarding their daily and end-of-life care [2, 3]. Without prior discussion about future care preferences, the concordance between people with dementia and their family members tends to be low [4]. Surrogate decisions impose psychological and decisional burdens on the family members [5, 6]. Hence, surrogate medical decisions are more likely to result in futile and aggressive life-sustaining treatments at the end-of-life stage for people with dementia [7].

Advance care planning (ACP) is a continuous, dynamic process that helps an individual to understand their personal values, life goals, and preferences for future medical treatment and care and to discuss these with their family and healthcare providers [8, 9]. Early initiation of ACP for people with dementia has been widely advocated [8, 10, 11], as this can promote their involvement in their own future care planning when they still have sufficient mental capacity. Nevertheless, few ACP interventions to involve people with early-stage dementia are available [12–14]. Most of the few available interventions are multi-component psychosocial interventions in which ACP is embedded; few studies of these have reported on ACP engagement using validated instrument [15–19], and few studies have been underpinned by theoretical framework [20, 21]. Hence, conclusions regarding the mechanisms and effects of these interventions on ACP engagement have yet to be drawn.

Furthermore, most studies on ACP interventions have been conducted in Western countries; therefore, little is known about culturally appropriate strategies to engage people with dementia in ACP in the Chinese context. In Chinese society, discussion about end-of-life care issues is a cultural taboo [22, 23]. An individual’s right to decision-making about health care is usually overridden by medical authority [22], or family [3, 24–26].

Following the recommendations of the Medical Research Council on developing and evaluating complex interventions [27], we developed a culturally appropriate dyadic ACP intervention – the ‘Have a Say (HAS)’ programme – for Chinese people with early-stage dementia. First, we performed an integrative review that revealed a lack of knowledge about dementia, limited awareness and support of ACP, and fear of provoking negative emotions as barriers to ACP engagement for people with early-stage dementia [28–31]. Second, we conducted a qualitative study that suggested that the dyadic participation of people with dementia and their family members, peer learning, trusting relationships between the dyads and ACP facilitators, and a person-centred approach were enablers to engaging people with early-stage dementia in ACP in the Chinese context. Therefore, we developed an ACP programme that incorporates the enablers identified in our qualitative study, aiming at promoting self-efficacy in ACP among people with early-stage dementia. The HAS programme, underpinned by the Bandura’s self-efficacy model [32], encompasses four sources of self-efficacy in its components: (1) *Vicarious experience:* participants learn the concept and benefits of ACP by watching micro-movies on the life-stories of two persons with dementia, one who has completed ACP and one who has not; (2) *Verbal persuasion:* participants receive verbal information about the dementia trajectory, future care needs, surrogate role in decision-making and advance directives, and encouragement for participating in ACP discussions; (3) *Emotional arousal:* participants are supported by ACP facilitators to express their feelings and concerns throughout the ACP process; and (4) *Performance accomplishment:* participants are actively engaged and guided by the ACP facilitators to express their care preferences. Under this framework, the HAS programme enables participants with early-stage dementia to ‘have a say’ in their own future care by providing them with adequate knowledge, awareness, and opportunities to discuss ACP with their family caregivers and healthcare professionals in a supportive atmosphere.

## Methods

### Aim and objectives

This study was a feasibility trial, the results of which were used to inform a subsequent definitive pragmatic randomised controlled trial. The primary objectives of this feasibility trial were as follows:

1. To assess the feasibility of intervention procedures and of subject recruitment in terms of the recruitment rate;
2. To assess the feasibility of subject retention in terms of the attrition rate at all time points;
3. To identify participants’ reasons for refusal to participate and dropout throughout the study period; and
4. To assess the acceptability of the intervention to participants with early-stage dementia and their family caregivers in terms of the completion rate and satisfaction score.

This study also qualitatively explored the ACP facilitators’ experiences of implementing the intervention and the dyads’ experiences of participating in the intervention and identified improvement strategies from both stakeholders to inform the definitive trial.

The secondary objectives of this feasibility trial were as follows:

1. To assess the average duration required to deliver the intervention in a real community care setting;
2. To assess the feasibility of the study instruments in measuring the study outcomes in terms of data completeness for a subsequent definitive trial; and
3. To estimate the potential effects of the intervention.

### Study design

Initially, this was a multi-centre randomised controlled trial in which randomisation was performed at the centre level, and subject recruitment was started after randomisation. This study was conducted from September 2019 to March 2020. During the recruitment period, Hong Kong was undergoing a period of social unrest, during which the study sites (i.e., the elderly community centres) encountered unprecedented service disruption. It was expected that randomisation at the centre level would add uncertainty to intervention site retention, subject recruitment, and study implementation, which made the original intervention protocol impractical under this period of unrest. We therefore amended the protocol to a quasi-experimental, pretest–posttest study with a control group. The amended study reported here was in compliance with the Declaration of Helsinki and is retrospectively registered on 14/08/2020 at clinicaltrials.gov (Identifier: NCT04513106). This study is reported in accordance with the Consolidated Standards of Reporting Trials (CONSORT) guidelines for pilot and feasibility trials [33].

### Study settings

This study was conducted in elderly community centres in Hong Kong, which are publicly funded to provide social care and recreational activities to community-dwelling older adults and their family members.

### Participants

Eligible participants were Cantonese-speaking Chinese adults who i) were aged 55 years or above, ii) were at stage 3 or 4 on the Global Deterioration Scale [34], and iii) had a designated family member willing to participate in this study. People with dementia were assumed with full mental capacity according to recommendations for ACP in dementia [11] unless they were unable to communicate and comprehend adequately the information for informed consent process during subject recruitment. They were excluded if they i) had undergone ACP, ii) had previously signed an advance directive, or iii) had a terminal illness with a life expectancy of less than 6 months. The inclusion criteria for the designated family caregiver were as follows: they were i) nominated by the participants with early-stage dementia and were involved in making care- and medical-related decisions for him/her, ii) aged 18 years or above, and iii) able to speak Cantonese. Paid caregivers were excluded.

### Sample size justification

The sample size for this feasibility trial was calculated based on the effect size of the primary outcome of this study, ACP engagement level, among community-dwelling older adults in a randomised controlled trial [35]. The findings showed that the effect size was 0.73 at 3 months post-intervention [35]. According to the stepped rules of thumb for pilot trial, 10 participants is required per group for an intervention with an effect size larger than 0.7 to attain a power of 80% at a significance level of 5% [36]. We took into account an attrition rate of 34.3% reported by a local study involving Chinese patient–caregiver dyads in an ACP intervention [2]. Therefore, a total of 34 dyads is required, with 17 dyads per group.

### Agent-facilitated ACP intervention

Grounded in the literature [10, 11, 37] and our qualitative interviews with stakeholders on ACP (i.e. individuals with mild cognitive impairment or early-stage dementia, family caregivers of people with dementia, and healthcare staff working in dementia care), the HAS programme includes several features: 1) composed of multiple interactive sessions; 2) involving participants with early-stage dementia in the ACP process to respect their autonomy in decision-making; 3) involving family caregivers to facilitate family communication and preparing them to act as surrogates; 4) providing informational support about the trajectory of dementia; 5) supporting the dyads in decision-making; and 6) preparing frontline healthcare staff to implement the intervention and support ongoing discussion in their routine practice.

Dyads assigned to the intervention group received a group-based session and two individual dyadic-based sessions. Each session lasted approximately an hour was held on weekly basis to allow time for them to reflect on the content and have discussion. To ease the time and resource constraints posed by the intervention, in addition to the usual service in the real community care setting, the ACP facilitators provided flexibility for the dyads to schedule the two dyadic sessions within 1 month after the group-based session. Table 1 shows the programme outline. The purpose of the first session was to provide information about the disease trajectory of dementia, future care needs during advanced-stage dementia such as living arrangements, financial planning, life-sustaining treatment options at the end-of-life stage, surrogate decision-making, and advance directives. This session involved a didactic lecture, a micro-movie, video clips, and group discussions for the participants to exchange views. It was delivered by the first author who is a registered nurse with experience in end-of-life care and dementia care, has received training on ACP, and is one of the developers of the HAS programme.

**Table 1:** Outline of the ‘Have a Say (HAS)’ ACP programme

Week	Format	Objectives	Content
1	♦ A nurse-led group-based session ♦ Micro-movie and video watching ♦ Group discussion	♦ Increase participants’ understanding of dementia and advance care planning (ACP) ♦ Support participants to understand and share their views about future health decline ♦ Increase participants’ understanding of future care options ♦ Raise participants’ awareness of ACP	♦ Introduce the nature and trajectory of dementia and the care needs of people with dementia ♦ Share the life stories of two persons with dementia, one who has completed ACP and one who has not, through micro-movie ♦ Introduce information about ACP, including its rationale, contents, and benefits, using video clips ♦ Introduce various life-sustaining treatment options using video clips and demonstrate medical equipment, e.g. endotracheal tube and feeding tube ♦ Facilitate group discussion among participants to share their views about future health decline, end-of-life care preferences, and surrogate decision-making
2	♦ ACP facilitator-led dyadic sessions ♦ Individualised ACP discussion guided by an ACP booklet	♦ Explore participants’ values, preferences, and family resources and dyads’ concerns related to future care planning ♦ Provide information on various options for each future care issue ♦ Facilitate the discussion ♦ Support the dyads to make decisions about future care options ♦ Document participants’ views and preferences in the ACP booklet	♦ Discussion topics covered: - Personal values, wishes, and views about own health and future care - Place of care - Daily care arrangements - Feeding options
3			♦ Discussion topics covered: - Life-sustaining treatments at the end-of-life stage - Legal and financial arrangements - Surrogate decision-makers - Funeral arrangement - Other unfulfilled wishes

The subsequent two sessions were conducted on individual dyadic basis by an ACP facilitator, who was the case manager of the dyad at the participating centre. Such arrangement was informed by our qualitative study which revealed that an established rapport between the facilitator and dyads was one of the enablers of ACP implementation. Participants with early-stage dementia were guided to identify their personal values, concerns, and views about future care and communicate care preferences. Our team developed a dementia-specific ACP booklet which provides written information about the disease trajectory of dementia, potential benefits and harms of using life-sustaining treatments at end-of-life stage, and rationales of ACP. It also consists of a series of reflective questions to elicit personal values, views on current health status, life-sustaining treatments, and future care preferences. The ACP facilitators adopted the ACP booklet as conversation tool to guide the participants through the planning process, then documented their wishes and care preferences in the ACP booklets. To support ongoing discussions, the completed booklets were returned to the participants after completion of the programme. Participants were encouraged to review and update their views regularly and extend the conversation with their healthcare team.

To enhance intervention fidelity, an ACP facilitator guide was developed as reference for the intervention implementation. It detailed the key topics and purposes of each session of the intervention, the guiding questions, and the facilitation skills. A 3.5-hour ACP facilitator training workshop was arranged at the study sites allocated to the intervention group. Staff members who were social workers, nurses, or allied health professionals and had more than 2 years’ experience in providing elderly care services were included in this training workshop led by the first author. The training contents covered ACP concepts, the role of the ACP facilitators, and ACP facilitation skills, which are detailed in Table 2. The third author who has extensive experience in developing and implementing ACP interventions and capacity building in dementia care was actively involved in the development of training materials [2, 38]. She also attended the first two training workshops and provided immediate feedbacks to the first author to ensure the validity of the training. To monitor the intervention fidelity, the first author also randomly attended at least one dyadic session conducted by each ACP facilitator to monitor their facilitation skills and ensure protocol adherence. The first author provided feedback to each of the ACP facilitators through individual debriefing meetings and maintained contact with them to provide support throughout the study period.

**Table 2:** Outline of the ACP facilitator training workshop

Topic	Learning Outcome
What is ACP?	Define the concept of ACP and the local policy on ACP and advance directives
Why people with dementia need ACP?	Understand the relevance and significance of ACP for people with dementia and recommendations for healthcare professionals
What are the roles of ACP facilitators?	Understand the roles of ACP facilitators: initiating ACP discussion, providing informational support, facilitating shared decision-making, and recording and reviewing ACP discussion
How to initiate ACP? - Contents	Identify the contents to be discussed with individuals with dementia: - dementia trajectory; - health needs and daily care arrangements at the late stage of dementia; - role of surrogate decision-makers; - preference of life-sustaining treatments at the end-of-life stage; - preference of financial and funeral arrangements; - values, wishes, and goals of care
- Facilitation skills	Get familiar with the facilitation skills to initiate ACP with individuals with dementia: - the attitude of ACP facilitators; - the barriers to ACP engagement among individuals with dementia; - the principles of considering the mental capacity of individuals with dementia; - consideration of the participants’ readiness for ACP; - the process of ACP guided by an ACP booklet

### Attention control

Dyads in the attention control group received three sessions consisting of educational health talks related to ageing that were neither dementia-specific nor related to ACP. The sessions, with each lasted approximately 1 h, were held on a weekly basis to equalize the contact time and attention given to the participants in the intervention group.

### Group allocation

The participating elderly community centres were pair-matched based on their scope of service and scale. The two centres from each pair were assigned to the intervention group or the control group at a 1:1 ratio by an independent research assistant. Only the superintendents of the participating centres were notified about the group allocation results; the staff members who assisted in subject recruitment and a research assistant who conducted outcome assessments were blinded to group allocation.

### Outcome measures

#### Feasibility of the trial design

The intervention site recruitment rate was measured as the proportion of invited centres that agreed to participate, while the subject recruitment rate was measured as the proportion of subjects referred by the centres who were eligible and consented to join the trial. The attrition rates of the study sites and subjects were calculated, and the reasons for attrition, such as refusal, withdrawal, and dropout, were obtained.

#### Acceptability of the intervention

The acceptability of the intervention was determined by the completion rate and satisfaction scores of the participants. The completion rate was calculated as the proportion of participants who attended at least two of the three sessions. The intervention group participants were asked to complete a self-developed 5-item satisfaction questionnaire to provide ratings on the content, timing, and duration of the intervention; the usefulness of the information provided; and the performance of the ACP facilitators. The items were rated on a 5-point Likert scale (5 = totally agree; 1 = totally disagree), with a higher total score indicating greater satisfaction with the intervention.

#### Average duration of the intervention

The number of weeks between the first and last sessions was recorded to determine the average duration required to deliver the intervention in the real community care setting.

#### Feasibility of the study instruments

The feasibility of the study instruments, listed in the following, for outcome evaluation was determined based on the instrument completion rate at different time points.

- *ACP Engagement Survey.* The 9-item version comprised two subscales, one each measuring self-efficacy and readiness for ACP engagement [35]. Its Chinese version has been validated with sound psychometric properties [39]. Each item is scored from 1 to 5, with a higher total score representing a higher level of ACP engagement. Those who did not respond according to the response format and chose the “uncertain” or “refuse” response, was counted as invalid response. During data analysis, these responses were replaced by “1” which indicated “not confident at all” in the self-efficacy subscale or “I have not thought about this before” in the readiness subscale.
- *Life-Support Preferences Questionnaire.* This was used to measure the concordance between the end-of-life care preferences of participants with early-stage dementia and those predicted by their family caregivers [40]. This questionnaire was previously modified on the basis of the local advance directive form and used in our studies with frail older adults in hospitals and old age homes, seriously ill patients and their family members in the Chinese context [2, 41, 42]. The questionnaire assesses preferences regarding (i) three kinds of life-sustaining treatments (cardiopulmonary resuscitation, mechanical ventilator, and tube feeding) based on three options (want to attempt, refuse or uncertain) and (ii) care goals (comfort-oriented, prolongation of life at all costs or uncertain) regarding two hypothetical end-of-life scenarios (terminally ill and in a persistent vegetative state or irreversible coma). Dyadic concordance on each treatment decision was determined by whether the dyad chose the same response, regardless that was for active treatment or not, they gained a score of one. Given the nature of ACP is for the dyad to communicate preferences on end-of-life care, no score would be given if anyone in the dyad chose the “uncertain” response. The total concordance score ranges from 0 to 8, with a higher score representing a higher dyadic concordance.
- *Cornell Scale for Depression in Dementia.* This is a 19-item instrument used to measure the signs and symptoms of depression in participants with early-stage dementia [43]. Its Chinese version was reported to have sound psychometric properties [44]. Each item is scored from 0 to 2, with a higher score representing a higher level of depression, and a cut-off of 8 indicates mild depression and 12 for major depression [43].
- *Zarit Burden Interview.* This is a 12-item instrument used to measure caregivers’ stress [45]. Its Chinese version was reported to have sound psychometric properties [46]. Each item is scored from 0 to 4, with a higher score representing a higher level of caregiver burden.

### Process evaluation

Individual semi-structured interviews were conducted with a purposive sample of participants in the intervention group, including participants with early-stage dementia with different age, sex, stage of Global Deterioration Scale, and living status, and their family caregivers in different capacity. ACP facilitators who delivered the dyadic sessions to two or more dyads were also invited to share their experiences of implementing the intervention. Data collection ceased upon data saturation, i.e., when no new ideas emerged. Interviews were conducted by the first author in private single rooms at the participating centres or at the participants’ homes. An interview guide was developed to assist the author in conducting the interviews (Table 3).

**Table 3:** Semi-structured interview guide

**Interview guide for ACP facilitators**
What is your experience with subject recruitment? What is your perception of the ‘**Have a Say (HAS**)’ **programme**? What are the best features of this programme? What are the worst features of this programme? What are the facilitators of and barriers to implementing this programme? What are your suggestions to improve this programme?
**Interview guide for participants**
What is your perception of the **HAS programme**? What are the best features of this programme? What are the worst features of this programme? What has helped you to engage/inhibited you from engaging in ACP throughout the programme? What are your suggestions to improve this programme?

### Prespecified criteria for progress to the definitive trial

Based on the study objectives to test the feasibility and acceptability of the intervention, the following progression criteria must be met to indicate the feasibility of proceeding to the definitive trial:

1. A completion rate for intervention and instrument of 80%; and.
2. An average satisfaction score of 4 out of 5.

### Subject recruitment

The staff members of each participating centre reviewed their client database, identified the potential participants, and introduced the study to them through phone or face-to-face contact. Those who were interested in the study were referred to the research team. The first author then screened the eligibility of the potential participants in person and explained the study details to those who were eligible to participate. Written consent to participate was sought from eligible participants with early-stage dementia and their family caregivers.

### Study procedures

The sociodemographic data (age, sex, living status, educational status, working status, financial status, and religion) of the participating dyads were collected by a trained research assistant. The cognitive functioning of the participants with dementia was assessed using the Montreal Cognitive Assessment (Chinese version). The study instruments were administered to the participants at baseline (T0), immediately after the intervention (T1), and at 1 month (T2) and 3 months (T3) post-intervention. The participants from the intervention group were also asked to complete the satisfaction questionnaire at T1. Each participant received a HK$100 (approximately US$13) cash voucher upon completion of the study to compensate for the cost of travel to the study sites.

### Ethical considerations

This study was approved by the Joint Chinese University of Hong Kong – New Territories East Cluster Clinical Research Ethics Committee, and the Research Ethics Committee of the Hong Kong Metropolitan University (Approval no. 2019.438 and HE-OT2019/03, respectively). Participation was voluntary, and the participants could withdraw from the study at any time without any consequences for the usual care they received.

### Data analysis

Quantitative data were analysed using the IBM SPSS Statistics version 26.0. Descriptive statistics were used to present the sociodemographic and clinical characteristics of the participants, recruitment rate, intervention completion rate, participant satisfaction scores, attrition rate, and missing rate for study instruments at various time points. The homogeneity of the participants in the two study groups was examined by comparing their characteristics using Chi-square test for categorical variables and the Mann–Whitney U test for continuous variables. Any significant group differences were identified as covariates and adjusted in the outcome analysis. Generalised estimating equations (GEE) were employed to examine the differential changes in study outcomes at T1, T2 and T3 with respect to T0. Effect size was measured by dividing the mean difference between two groups by the pooled standard deviation and reported as Cohen’*d* value. The cut-off points of small, medium and large effect size measured by Cohen’*d* are 0.2, 0.5 and 0.8, respectively [47]. All analysis was considered significant at *p* value less than 0.05 (2-tailed).

All interviews were audio-recorded and transcribed verbatim for qualitative content analysis [48]. The authors familiarised themselves with the transcripts by reading and re-reading them. They extracted the meaning units by focusing on both manifest and latent content. Manifest content refers to visible and obvious components of the text, whereas latent content involves an interpretation of the underlying meaning of the text. Each author performed a preliminary analysis of the data and developed an initial coding framework independently. They discussed the coding process and revisited the categories and subcategories until a consensus was reached. Illustrative quotes were selected through critical discussion between authors. The first author translated the quotes to English while the other authors helped with verification and proofreading.

## Results

### Study site recruitment

Six elderly community centres run by different operators – four at the district level and two at the sub-district level – agreed to participate in this study. They were pair-matched on the basis of the service scale and assigned to either the intervention group or the control group. One centre at the sub-district level in the intervention group withdrew from the study because the staff members could not identify any potential participants among their existing clients and the superintendent worried that it would be time-consuming to open up subject recruitment for outsiders.

### Subject recruitment

Subject recruitment spanned from September 2019 to October 2019, until the estimated sample size was reached. Five centres identified 60 dyads as potential participants, ranging from 4 to 18 dyads per centre. Of them, one dyad did not meet the inclusion criteria and 23 dyads refused to participate, giving a recruitment rate of 60%. The major reasons for refusal were lack of interest in ACP among participants with dementia (*n* = 12) or family caregivers (*n* = 4), scheduling conflicts with other activities for participants with dementia (*n* = 3), or lack of time among family caregivers (*n* = 4). Eventually, 36 dyads participated in the study, with 18 per group.

### Characteristics of the participants

Table 4 shows the sociodemographic and clinical characteristics of the participants. Of the 36 participants with dementia, their mean age was 82.7 years (SD = 6.9, ranging from 62 to 94). Over half of them were male (55.6%), had no formal education (55.6%), and had a Global Deterioration Scale at stage 4 (52.8%). The mean age of their family caregivers participating in this study was 64.2 years (SD = 13.3, ranging from 30 to 85). Significantly more participants in the intervention group than the control group were living alone (*p* = 0.003) and at stage 3 of Global Deterioration Scale (*p* = 0.019). For the family caregivers, most of them were female (83.3%) and had education beyond the secondary school level (69.4%). Compared with the control group, family caregivers in the intervention group were significantly younger (*p* = 0.030) because a larger proportion of them were adult children (*p* = 0.048), and with a significantly lower proportion was living with the participants (*p* = 0.044).

**Table 4:** Participants’ characteristics

	ALL (*N* = 36)	Intervention (*n* = 18)	Control (*n* = 18)	*p*
**Participants with early-stage dementia**				
*Age (years)^*	82.7 (6.9)	81.6 (8.6)	83.8 (4.6)	.340
*Sex*				.502
Male	16 (44.4%)	7 (38.9%)	9 (37.5%)	
Female	20 (55.6%)	11 (61.1%)	9 (37.5%)	
*Marital Status*				.216
Married/cohabiting	24 (66.7%)	10 (55.6%)	14 (77.8%)	
Widowed	10 (27.8%)	6 (33.3%)	4 (22.2%)	
Separated/divorced	2 (5.6%)	2 (11.1%)	0	
*Educational level*				.745
No formal education	20 (55.6%)	11 (61.1%)	9 (50%)	
Primary school	5 (13.9%)	3 (16.7%)	2 (11.1%)	
Secondary school	8 (22.2%)	3 (16.7%)	5 (27.8%)	
Tertiary education or above	3 (8.3%)	1 (5.6%)	2 (11.1%)	
*Living status*				.003
Living alone	10 (27.8%)	9 (50%)	1 (5.5%)	
Living with others	26 (72.2%)	9 (50%)	17 (94.4%)	
*With religious belief*				.502
Yes	23 (63.9%)	12 (70.6%)	11(61.1%)	
No	12 (33.3%)	5 (29.4%)	7 (38.9%)	
*Received government allowance*				.310
Yes	35 (97.2%)	17 (94.4%)	18 (100%)	
No	1 (2.8%)	1 (5.6%)	0	
*Global Deterioration Scale*				.019
Stage 3	17 (47.2%)	12 (66.7%)	5 (27.8%)	
Stage 4	19 (52.8%)	6 (33.3%)	13 (72.2%)	
*Montreal Cognitive Assessment score^*	14.4 (4.5)	15.4 (4.2)	13.6 (4.7)	.067
**Family caregivers**				
*Age (years)*^	64.2 (13.3)	59.4 (13.2%)	68.9 (11.9%)	.030
*Sex*				.371
Male	6 (16.7%)	2 (11.1%)	4 (22.2%)	
Female	30 (83.3%)	16 (88.9%)	14 (77.8%)	
*Marital Status*				1.000
Married/cohabiting	28 (77.8%)	14 (77.8%)	14 (77.8%)	
Single/Divorced/Widowed	8 (22.2%)	4 (22.2%)	4 (22.2%)	
*Educational level*				.952
No formal education	7 (19.4%)	3 (16.7%)	4 (22.2%)	
Primary school	4 (11.1%)	2 (11.1%)	2 (11.1%)	
Secondary school	20 (55.6%)	10 (55.6%)	10 (55.6%)	
Tertiary education or above	5 (13.9%)	3 (16.7%)	2 (11.1%)	
*Living status*				.044
Living with the participant	20 (55.6%)	7 (38.9%)	13 (72.2%)	
Not living with the participant	16 (44.4%)	11 (61.1%)	5 (27.8%)	
*Working status*				.151
Full-time employment	9 (25.0%)	7 (38.9%)	2 (11.1%)	
Part-time employment	3 (8.3%)	1 (5.6%)	2 (11.1%)	
Unemployed	24 (66.7%)	10 (55.6%)	14 (77.8%)	
*Relationship with the participant*				.048
Spouse	15 (41.7%)	4 (22.2%)	11 (61.1%)	
Child	20 (55.6%)	13 (72.2%)	7 (38.9%)	
Others	1 (2.8%)	1 (5.6%)	0	

Footnote: Data marked with ^ are presented as the mean (standard deviation); otherwise frequency (%)

### Subject retention

**Fig. 1:**
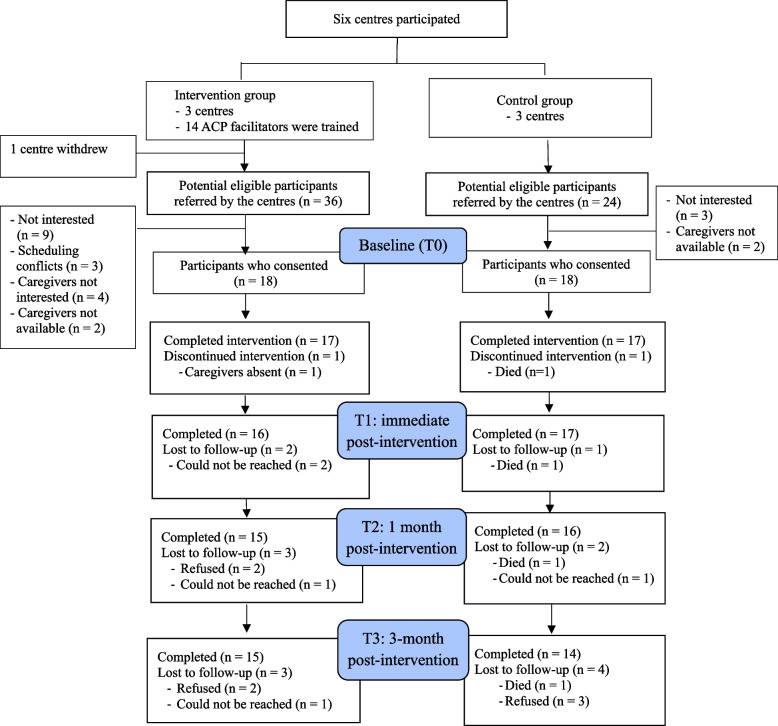
Flow diagram of the study

The attrition rates at T1, T2, and T3 were 8.3%, 13.9%, and 19.4%, respectively (Fig. 1). Seven participants with dementia withdrew from the study due to several reasons, including one died of acute illness at T1; two were annoyed with questionnaire administration (*n* = 2 at T2 and T3); two could not be reached (*n* = 2 at T1 and T2); and three in the control group could not recall their participation in the study at T3. Two participants found the questionnaire about end-of-life issues distressing, particularly around the time of Lunar New Year. For those who could not be reached, they were either hospitalised or their family caregivers worried that the questionnaire would arouse negative emotions.

### Acceptability of the intervention

Except for one family caregiver being absent from all intervention sessions due to scheduling conflicts, all participants attended all intervention sessions, giving a dyadic completion rate of 94.4%. All participants assigned to the intervention group responded to the satisfaction survey, and the mean overall satisfaction score was 4.4/5, with mean scores of 4.4 for intervention content, timing of introducing ACP, and duration of the sessions and 4.5 for the usefulness of information and the performance of the ACP facilitators. We did not receive any report of adverse events related to the intervention.

Qualitative interviews were conducted with four dyads of participants and four ACP facilitators. Their demographic characteristics are shown in Table 5. Two categories concerning the strengths of the intervention and implementation challenges were identified from the interviews. The sub-categories within each category are shown in Tables 6 and 7.

**Table 5:** Characteristics of informants in qualitative interviews

Participants with dementia				
Code	Sex	Age (years)	GDS stage	Living status
A02	Female	83	3	Live alone
A07	Female	93	3	Live alone
B01	Female	71	4	Live with others
B06	Male	84	4	Live with others
Family caregivers of participants with dementia				
Code	Sex	Age (years)	Relationship	Living with participants with dementia
A02_CG	Female	58	Child	No
A07_CG	Female	70	Child	No
B01_CG	Female	46	Child	No
B06_CG	Female	82	Spouse	Yes
Advance care planning facilitators				
Code	Sex		Profession	Working experience
Staff A	Female		Social worker	< 5 years
Staff B	Female		Social worker	5–10 years
Staff C	Female		Social worker	10–15 years
Staff D	Female		Social worker	10–15 years

*GDS* Global Deterioration Scale

**Table 6:** Qualitative findings

Category	Sub-category
Strengths of the intervention	Triadic involvement
	Trusting relationship
	Documentation of client’s views and care preferences
Implementation challenges	Structured format of the intervention
	Discussing medical issues

**Table 7:** Between-group comparisons of outcome variables across three time points and the effect size

Outcome	Mean (SD)		Group effect		Time effect		Group*Time effect		Effect Size (*d*)
	Intervention	Control	β (95% CI)	*p*	β (95% CI)	*p*	β (95% CI)	*p*	
ACP Engagement: Self-efficacy subscale									
T0	3.57 (1.28)	3.13 (1.14)	0.44 (-0.33, 1.22)	.259		-	-	-	-
T1	4.13 (1.23)	3.00 (1.05)			-0.12 (-0.89, 0.58)	.741	0.63 (-0.27, 1.53)	.170	0.99
T2	4.02 (0.89)	3.04 (1.34)			-0.14 (-0.88, 0.61)	.723	0.57 (-0.40, 1.53)	.253	0.86
T3	4.00 (1.29)	2.52 (1.69)			-0.74 (-1.63, 0.15)	.105	1.16 (0.11, 2.20)	.030	0.98
ACP Engagement: Readiness subscale									
T0	1.74 (1.08)	1.06 (0.13)	0.68 (0.19, 1.16)	.007	-	-	-	-	-
T1	2.49 (1.00)	1.20 (0.28)			0.14 (-0.01, 0.28)	.070	0.54 (0.15, 0.92)	.006	1.76
T2	2.13 (0.99)	1.41 (0.50)			0.34 (0.10, 0.57)	.006	-0.02 (-0.52, 0.47)	.925	0.92
T3	2.24 (1.17)	1.18 (0.34)			0.06 (-0.14, 0.27)	.538	0.38 (-0.06, 0.82)	.088	1.23
Life Support Preferences Questionnaire									
T0	3.61 (2.68)	3.39 (3.22)	-0.94 (-2.61, 0.72)	.267	-	-	-	-	-
T1	7.00 (1.37)	3.29 (2.71)			0.23 (-1.13,1.59)	.744	2.83 (1.02, 4.65)	.001	1.73
T2	6.07 (2.02)	2.69 (3.03)			-0.22 (-1.86, 1.45)	.805	2.49 (0.28, 4.69)	.027	1.31
T3	6.40 (2.44)	3.14 (3.11)			-0.97 (-2.54, 0.60)	.224	3.64 (1.52, 5.75)	.002	1.17
Cornell Scale for Depression in Dementia									
T0	5.17 (4.63)	6.28 (6.36)	-1.11 (-4.64, 2.42)	.538	-	-	-	-	-
T1	4.06 (4.77)	3.29 (2.27)			-2.97 (-5.98, 0.04)	.053	1.76 (-1.74, 5.26)	.324	0.21
T2	3.20 (2.27)	2.56 (2.66)			-3.64 (-6.99, -0.29)	.033	1.54 (-2.44, 5.52)	.449	0.26
T3	2.53 (3.31)	3.07 (2.87)			-3.15 (-5.88, -0.41)	.024	0.48 (-2.96, 3.91)	.786	0.17
Zarit Burden Interview									
T0	10.67 (5.64)	12.22 (10.58)	-1.56 (-6.94, 3.83)	.571	-	-	-	-	-
T1	11.25 (7.50)	9.82 (8.24)			-2.94 (-5.96, 0.09)	.057	3.23 (-0.41, 6.86)	.082	0.18
T2	11.07 (7.12)	7.44 (5.69)			-5.50 (-9.72, -1.27)	.011	4.97 (0.23, 9.72)	.040	0.56
T3	8.53 (8.50)	8.64 (6.08)			-3.90 (-8.15, 0.35)	.072	1.07 (-4.33, 6.47)	.697	0.01

Generalized estimating equations; T0: baseline assessment; T1: immediately post-intervention; T2: 1-month post-intervention; T3: 3-month post-intervention; adjusted for living status and Global Deterioration Scale of participants with dementia

#### Category 1: Strengths of the intervention

- *Triadic involvement*

Both the participants and ACP facilitators appreciated the opportunity to bring the clients, family caregivers, and healthcare providers together to openly discuss future care. It enabled the participants to clarify their views and concerns with the healthcare provider and bridge communication gaps within the family.

A participant with dementia shared,

‘I think it is good. I can understand different situations and procedures. I can make my choice. In the future, my daughters need not argue.’ (A02)

A family caregiver stated,

‘I can search this information from the Internet myself, but through this programme, I can learn and ask questions in-person. I can know much more.’ (A02_CG)

The sharing of another family caregiver further underscored the importance of having an ACP facilitator to bring up the topic for discussion. She said,

‘This programme is very good for the caregivers. We won’t think about or raise such discussions ourselves because [we worried that] mum may not want to discuss these issues. We are uncertain of her responses… Now, we can have the discussion in a relaxed atmosphere, and we can express our views.’ (B01_CG)

An ACP facilitator highlighted the importance of involving family members in the discussion as she noted,

‘The family caregivers can help to convey their [participants with dementia] views to the whole family.’ (Staff C)

- *Trusting relationships*

The rapport between the facilitators and participants was identified by the participants and ACP facilitators as a cornerstone for the potentially sensitive discussion. The facilitators found that their established relationships with the dyads were imperative to initiate the conversation.

One ACP facilitiator stated,

‘Older people usually have boundaries. Not everyone can engage them to talk about this. It must be one who is familiar with and knows them well.’ (Staff D)

A participant with dementia also shared,

‘She [the ACP facilitator] always takes good care of me. She helps me to arrange all I need. Like this activity, I believe she is doing good to me.’ (A07)

- *Documentation of clients’ views and care preferences*

The participants and facilitators recognised the need for documenting the views and preferences identified during the planning process in the ACP booklet to facilitate continual discussions over time, particularly for some participants who reported difficulty in recalling the details of the ACP conversation. A family caregiver shared,

*‘The booklet is useful. I treat it as a treasure. It has notes on important issues about my husband. We cannot recall them without the booklet. I will pass it to my children.’ (B06_CG)*

In addition, the ACP facilitators found the ACP booklet useful as a conversation guide. One of them stated,

‘The ACP booklet is useful to guide the ACP discussion. It enhanced my confidence to initiate the discussion.’ (Staff A)

#### Category 2: Implementation challenges

- *Structured format of the intervention*

The applicability of the structured format of the intervention was questioned by the participants. The ACP facilitators reported that the pace of discussion varied among the participants; for example, some participants required extra time, particularly if they were experiencing emotional distress. Two facilitators shared,

‘Some participants take longer time to understand and require more elaboration, especially on topics that are new to them.’ (Staff B)

‘For those who are not feeling good, you have to handle their emotions before moving on to the discussion. It imposes a time pressure.’ (Staff D)

Another facilitator noted that the two dyadic sessions could be combined. She shared,

*‘Both of my cases went well with the flow. It takes time to set the scene and recall their memory at the beginning of each session. Breaking the discussion into two sessions requires extra effort to create the atmosphere again and is not necessary.’ (Staff C)*

This facilitator also pointed out the challenge of scheduling the sessions with the participants. She stated,

‘It takes me a lot of effort to schedule the individualised sessions with the dyads. We have to compromise among three parties.’ (Staff C)

One family caregiver also highlighted the time commitment required for participation. She shared,

*‘I understand that we need such a long time to go through the ACP process in detail. But to most of the caregivers, especially those who need to work, attending all three sessions may not be possible.’ (A07_CG)*

- *Discussing medical issues*

Discussion on the ACP process was largely grounded in the disease trajectory of dementia and the potential medical treatments that could be used in future care. The participants required much informational support for medical decision-making. A facilitator who is a social worker found it difficult to explain the information and provide decisional support due to their limited knowledge on medical issues. She stated,

‘When they ask about resuscitation procedures and outcomes, we don’t have the clinical experience or knowledge to answer.’ (Staff A)

### Average duration of the intervention

All dyadic sessions were delivered within 3 to 8 weeks after the first group-based session. The average duration to complete the intervention was 5.4 weeks (SD = 1.45).

### Feasibility of the study instruments

The participants were able to complete all study instruments except the ACP engagement survey. The proportion of invalid responses among participants with dementia in the self-efficacy subscale of the ACP engagement survey at T0, T1, T2, and T3 were 13.9%, 21%, 19.8%, and 26.4% respectively, and that in the readiness subscale were 1.9%, 0%, 10.9%, and 10.9%, respectively.

### Potential effects of the HAS programme

The participants with dementia in the intervention group reported a significantly greater improvement, with large effect sizes, in self-efficacy for ACP at T3 (*p* = 0.030;* d* = 0.98) and readiness for ACP at T1 (*p* = 0.006;* d* = 1.23), compared with those in the control group. The between group differences in depressive symptoms were not significant across time. Family caregivers of participants in the intervention group reported a significantly higher level of caregiving burden, with medium effect size, at T2 (*p* = 0.040;* d* = 0.56) than those in the control group. Regarding the dyadic concordance on end-of-life care preference, the intervention group demonstrated significantly greater improvement, with large effect sizes, at all time-points (Group*T1: β = 2.83, *p* = 0.001; Group*T2: β = 2.49, *p* = 0.027; Group*T3: β = 3.64, *p* = 0.002) (Table 7).

## Discussion

We are the first to develop a theory-guided dyadic ACP intervention for people with early-stage dementia and their family caregivers in a Chinese community care setting. In this feasibility study, the trial design was found to be feasible in real community care settings, with a recruitment rate of 60%, an intervention completion rate of 94.4%, and an attrition rate of 19.4% at 3 months post-intervention. The intervention was also acceptable to the participants, as suggested by their high satisfaction scores and qualitative comments.

Our study has two strengths. Firstly, the HAS programme was developed according to the guidelines on designing and evaluating complex interventions established by the Medical Research Council to overcome the sociocultural barriers to ACP engagement at individual and organisational levels [27]. The intervention content and format are designed on the basis of literature review, a theoretical framework, and qualitative findings from stakeholders to address the information and psychological needs of Chinese people with early-stage dementia and their family caregivers. Secondly, we adopted a clinician–researcher collaborative approach. We sought managerial support from elderly community centres to secure organisational support for ACP. We also provided ACP facilitator training to prepare frontline health and social care staff members to implement the intervention. This approach concords with previous expert consensus-based recommendations on ACP implementation to embed ACP in the organisational culture and equip frontline staff with the competence to initiate ACP [11, 49].

The subject recruitment in our study was completed within a shorter time (2 months) than that in previous studies, which required 20 to 24 months to achieve a similar sample size [17, 19]. Our subject recruitment rate at 60% was also higher than that reported in previous studies, which ranged from 15.8% to 27.9% [17, 21, 50]. The completion rate in our study (94.4%) was also higher than the rates reported in previous studies [19, 51]. The difference is likely due to the involvement of staff members at the participating elderly community centres in subject recruitment, who have established rapport with the potential participants. Most studies have recruited participants with early-stage dementia through postal mail invitation, which resulted in low response rates [21, 50]. Our qualitative findings also highlighted that triadic involvement and trusting relationships were key features being appreciated by the participants. These recruitment and intervention implementation strategies were in line with the recommendations for dyadic research [52].

Our findings demonstrated that the intervention design was acceptable to the participants in terms of its duration, format, and delivery approach, as suggested by the high completion rate and overall satisfaction score. Since dyadic interventions for participants with dementia and family caregivers with multiple sessions increase the participation burden [53], the HAS programme included a group-based and two individual sessions to balance between vicarious learning and personalisation in verbal persuasion for promoting self-efficacy. The ACP booklet as a guide for reflection and communication also enhance sense of accomplishment among the participants. The findings suggested that the HAS programme has the potential to enhance the participants’ readiness for ACP immediately after the intervention although the effects did not sustain, and the participants’ self-efficacy for ACP at 3 months after the programme. Echoed with our qualitative findings that time required for discussion greatly varied across the dyads, the results suggested the need of a booster session to clarify individual needs for ACP [11] so as to strengthen the intervention effect on ACP engagement.

The findings suggested that the HAS programme has large effect on improving the dyadic concordance regarding end-of-life care preferences at all follow-up time-points. This accords with an earlier study that mutual understanding can be achieved when both patient and family caregiver were involved in ACP [2]. Another important finding was that the HAS programme would not provoke depressive symptoms among participants with dementia. This could be attributed to the involvement of trusted healthcare staff as ACP facilitator and the arrangement of individualised sessions, which allow ACP facilitators to address participants’ personal emotional needs. However, a considerable proportion of invalid responses was noted in the ACP engagement scale, this raises the concerns on the validity of this instrument for people with dementia. One interesting finding was that family caregivers in the intervention group reported a significantly increased caregiving burden than the control group at 1 month after the programme, which contrasted to our hypothesis that family caregivers would experience less burden after the ACP intervention. However, the findings should be interpreted with caution due to the small sample size.

Nevertheless, several issues related to the implementation of our intervention were highlighted, which need to be addressed in the definitive trial. First, ACP facilitators who were social workers reported difficulties in discussing medical issues. Therefore, an additional nurse-led group-based session should be added to enhance the informational support to the participants for medical decision-making. Second, the time required for discussion greatly varied across the dyads. To accommodate individuals’ pace and schedules, flexibility in scheduling dyadic sessions within 6 weeks – the average duration to complete the HAS programme identified in this study – after the group-based session should be allowed in the definitive trial. Third, the proportion of invalid responses for the ACP engagement survey by participants with dementia was considerable across all time points. We recommend adding cognitive assessment of the participants with early dementia at each assessment time point to ensure their capacity to complete the instruments and also validate the instruments in this specific population. In addition, measuring the surrogate ACP engagement of family caregivers [54] can help to capture this outcome using a dyadic perspective. Fourth, significant differences in certain socio-demographic and clinical characteristics were noted between participants in the two groups. Apart from having a small sample size in this study, group allocation at centre level and without randomisation are other possible reasons. While we had pair-matched the centres in the two groups, the withdrawal of one centre in the intervention group might result in an imbalance in the participants’ characteristics. Therefore, randomisation at individual level would be a strategy to even out the sample characteristics between groups. Fifth, the timeframe for study should be cautiously planned. The data collection period in this study was close to the Chinese Lunar New Year, a festive time during which participants may be reluctant to discuss death and related issues, potentially decreasing their willingness to participate or respond to follow-up assessments. As ACP is a continual process of behavioural change and current evidence on the effects of ACP interventions on people with dementia over time is lacking, additional subject retention strategies should be attempted to minimise the attrition rate.

## Limitations

We acknowledge several limitations of this study. First, it may be subject to participation bias as the participants and staff members might have been more supportive of the idea of ACP than those who refused to participate. The participants were also more likely to have good family relationships than their non-participating counterparts given that they were required to join the study in dyads. It has been shown that dyads with better family relationships are more involved in end-of-life care issues [55]. Despite a purposive sampling was adopted in the qualitative interviews, the views from various group of informants were predominantly from the female perspectives due to voluntary participation. Second, the instruments have not been validated specifically among people with early dementia which might threaten the internal validity of the study. Third, the ACP facilitators in this study were predominantly social workers, and only one nurse was involved. It remains unknown whether the ACP facilitators’ experiences of implementing the intervention would be applicable to other allied health professionals. Forth, the satisfaction survey results could not reflect the acceptance of all interveners and the ACP booklet because the performance of the lecturer for the group-based session and the perceived usefulness of the booklet were not measured specifically. Lastly, the potential effects of the HAS programme were estimated from a small sample. A full-scale randomised controlled trial is warranted to examine the intervention effects. 

## Conclusions

In summary, this study provides preliminary evidence that a theory-guided ACP intervention is acceptable to both participants with dementia and frontline healthcare providers in the Chinese context, and that the trial design with a clinician–researcher collaborative approach is feasible in a real community care setting. Our findings suggest that the intervention warrants some modifications, an additional nurse-led group-based session on top of the original three sessions to discuss medical issues, and flexibility in scheduling individualised dyadic sessions. We also recommend that the ACP engagement levels of both participants with dementia and family caregivers be evaluated in the future definitive trial to compare the intervention effects between the dyadic members.

## Data Availability

The datasets used and/or analysed during the current study are available from the corresponding author on reasonable request.
